# Supplementary material for: The Caspase-3 homolog DrICE regulates endocytic trafficking during Drosophila tracheal morphogenesis
Source: Nat Commun. 2019 Mar 4;10:1031. doi: 10.1038/s41467-019-09009-z (PMC6399233; doi:10.1038/s41467-019-09009-z)
Supplement: Supplementary file 1 — Supplementary Information [file 41467_2019_9009_MOESM1_ESM.pdf]

The Caspase-3 homolog DrICE regulates endocytic trafficking during *Drosophila* tracheal morphogenesis

Saoirse S. McSharry1 and Greg J. Beitel1*

Key words: caspase; non-apoptotic; endocytic trafficking; morphogenesis; epithelial tube; Drosophila; trachea

1Department of Molecular Biosciences, Northwestern University, Evanston, IL 60208, USA

Running title: Caspase-mediated regulation of endocytic trafficking

* Author for correspondence and lead contact: Greg J. Beitel, Hogan Hall, Rm. 2-100, Northwestern University, Evanston, IL 60208, U.S.A., beitel@northwestern.edu, Ph: (847) 467-7776, FAX: (847) 467-1380

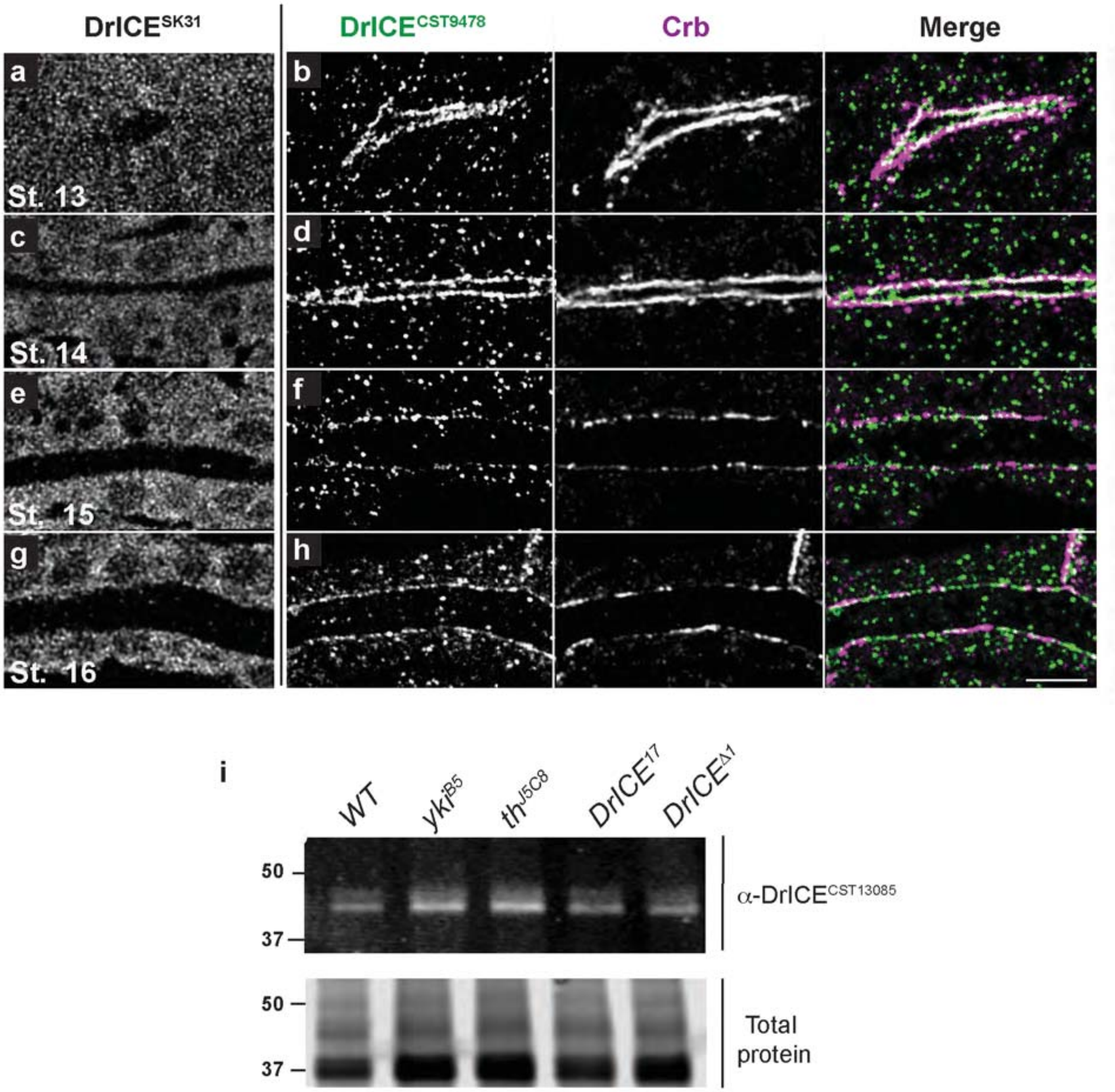

Supplementary Fig. 1: Developmental profile of DrICE with Crumbs, and DrICE levels in *yorkie*^{B5}. **(a-h)** The α-DrICE^{SK31} antibody against full-length DrICE[3] (a,c,e,g) reveals broad cytoplasmic staining during stages 13-16 (St.13- St. 16). Staining with the α-DrICE^{CST9478} antibody that was raised against a peptide that is cleaved during DrICE activation (Cell Signaling Technologies #9478) reveals a more restricted punctate pattern that is enriched at the tracheal apical surface (b, d, f, h; green) where it overlaps with staining for the apical marker Crumbs (Crb, b, d, f, h; magenta), particularly during tube expansion at stages 13 and 14 (b,d). Scale bar for a-h" in h", 5 μm.

(i) Western blot of stage 16 embryos using the α-DrICECST^{13085} antibody that recognizes full-length DrICE (Cell Signaling Technologies #13085). *yki*^{B5} and *th*^{J5C8} mutations appear to increase DrICE protein levels, but based on quantification of the 47kDa DrICE full-length band relative to total protein from three experiments, only the *yki*^{B5} difference is statistically significant (Fig. 1o). Note that DrICE protein levels in *DrICE*^{17} homozygotes are not different than WT, which is consistent with *DrICE*^{17} being a dominant negative allele that causes more severe tracheal phenotypes than the *DrICE*$^{\Delta 1}$ null allele.

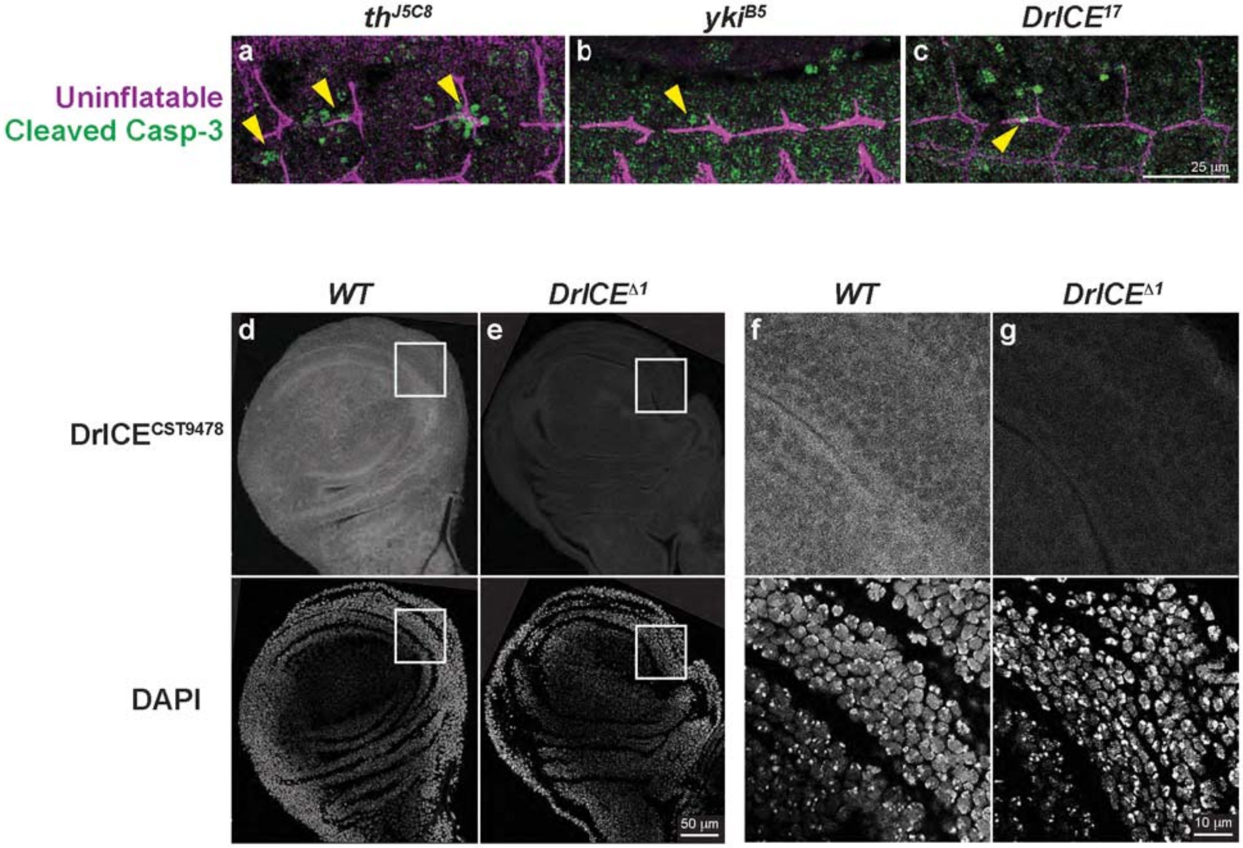

Supplementary Fig. 2: Cleaved caspase staining in Diap1/*th* embryos and *DrICE*$^{\Delta 1}$ imaginal discs. **(a-c)** Maximum projections of anti-cleaved caspase-3 staining, considered a marker of apoptotic cells,[4] shows that trachea of stage 13 *th*^{J5C8} embryos have noticeably more cleaved caspase-3 staining than either (b) *yki*^{B5} or (c) *DrICE*^{17} embryos. As thJ5C8 embryos also have a decreased total number of tracheal cells (Fig. 1m), these data support the conclusion that missing in the dorsal trunk segments in *th*^{J5C8} embryos (Fig. 1i) result from increased tracheal cell apoptosis. Scale bar for a-c in c, 25 µm.

(d-g) Staining WT (*w*^{1118}) late larval wing imaginal discs with DrICE^{CST9478} (low magnification view in e; magnification of the boxed region is shown in f) reveals a pattern of subcellular localization similar to embryonic tracheal cells. (e, g) DrICE^{CST9478} staining is absent in *DrICE*$^{\Delta 1}$ mutant wing discs, supporting the conclusion that the DrICE^{CST9478} signal present in *DrICE*$^{\Delta 1}$ mutant embryos (Fig. 2h) results from maternal contribution of DrICE. Bottom row shows DAPI staining of tissue. Scale bar for d- in bottom of e, 50 µm; for f-g in bottom of g, 10 µm.

Supplementary Table 1: Resources used in experimental analysis

REAGENT or RESOURCE	SOURCE	IDENTIFIER
**Antibodies**		
Guinea pig anti-uninflatable (1:800)	Rob Ward	
α-DrICE^{CST9478} Rabbit anti-cleaved Drosophila ICE Asp230 (1:100)	Cell Signaling Technology (CST)	9478S
α-DrICE^{CST13085S} Rabbit anti-Drosophila ICE (1:100)	Cell Signaling Technology (CST)	13085S
Mouse anti-Crumbs extracellular domain (1:25)	Developmental Studies Hybridoma Bank (DSHB) at the University of Iowa	Cq4-s
Mouse anti-2A12 (1:1)		
Rabbit anti-Serp (1:400)		
IRDye 800CW goat anti-Rabbit 0.5mg	Li-Cor	926-32211
Guinea-pig anti-DrICE (SK31)	Pascal Meier	SK31
Rat anti-Clathrin (Chc) (1:40)	Matthias Behr	n/a
Mouse anti-Rab5 (1:100)	BD Biosciences	610281
Mouse anti-Rab7 (1:50)	Developmental Studies Hybridoma Bank (DSHB) at the University of Iowa	Rab7
Mouse anti-Rab11 (1:100)	Fisher Scientific	BDB610656
Guinea pig anti-Melanotransferrin	Christos Samakovilis	n/a
Rabbit anti-Kune-Kune (1:500)	[1]	
Rabbit anti-Dlg (1:500)	Woods et al 1996[2]	
Goat anti-mouse IgG (H+L) highly cross-adsorbed secondary antibody Alexa Fluor 488 Plus	Life Technologies	A32723
Goat anti-mouse IgM (H+L) highly cross-adsorbed secondary antibody Alexa Fluor 488	Life Technologies	A10680
Goat anti-rabbit IgG (H+L) highly cross-adsorbed secondary antibody Alexa Fluor Plus 647	Life Technologies	A32733
Goat anti-guinea pig IgG (H+L) highly cross-adsorbed secondary antibody Alexa Fluor 568	Life Technologies	A11075
IRDye 800CW Goat anti-Rabbit 0.5 mg	Li-Cor	926-32211
**Critical Commercial Assays**		
REVERT Total protein stain kit	Fisher Scientific	NC1145693
Pre-cast gel 4-15%, 15 well	Bio Rad	4561086
**Software and Algorithms**		
Fiji (ImageJ) SpotCounter plugin		
Volocity Demo 5.5.1		
Leica Application Suite X (LAS X) HyVolution and colocalization calculation		
Li-Cor Image Studio Software		

References for Supplemental Figure Legends

1. Nelson, K.S., Furuse, M. & Beitel, G.J. The Drosophila Claudin Kune-kune is required for septate junction organization and tracheal tube size control. *Genetics* **185**, 831-839 (2010).
2. Woods, D.F., Hough, C., Peel, D., Callaini, G. & Bryant, P.J. Dlg protein is required for junction structure, cell polarity, and proliferation control in Drosophila epithelia. *The Journal of cell biology* **134**, 1469-1482 (1996).
3. Ditzel, M. *et al.* Inactivation of effector caspases through nondegradative polyubiquitylation. *Molecular cell* **32**, 540-553 (2008).
4. Baer, M.M. *et al.* The role of apoptosis in shaping the tracheal system in the Drosophila embryo. *Mechanisms of development* **127**, 28-35 (2010).
