# Supplementary material for: The Caspase-3 homolog DrICE regulates endocytic trafficking during Drosophila tracheal morphogenesis
Source: Nat Commun. 2019 Mar 4;10:1031. doi: 10.1038/s41467-019-09009-z (PMC6399233; doi:10.1038/s41467-019-09009-z)
Supplement: Supplementary file 3 — Reporting Summary [file 41467_2019_9009_MOESM3_ESM.pdf]

nature research

Corresponding author(s): Greg Beitel
Last updated by author(s): Dec 15, 2019

Reporting Summary

Nature Research wishes to improve the reproducibility of the work that we publish. This form provides structure for consistency and transparency in reporting. For further information on Nature Research policies, see Authors & Referees and the Editorial Policy Checklist.

Statistics

For all statistical analyses, confirm that the following items are present in the figure legend, table legend, main text, or Methods section.

n/a	Confirmed	
☐	☒	The exact sample size (*n*) for each experimental group/condition, given as a discrete number and unit of measurement
☐	☒	A statement on whether measurements were taken from distinct samples or whether the same sample was measured repeatedly
☐	☒	The statistical test(s) used AND whether they are one- or two-sided<br>*Only common tests should be described solely by name; describe more complex techniques in the Methods section.*
☐	☒	A description of all covariates tested
☐	☒	A description of any assumptions or corrections, such as tests of normality and adjustment for multiple comparisons
☐	☒	A full description of the statistical parameters including central tendency (e.g. means) or other basic estimates (e.g. regression coefficient) AND variation (e.g. standard deviation) or associated estimates of uncertainty (e.g. confidence intervals)
☒	☐	For null hypothesis testing, the test statistic (e.g. *F*, *t*, *r*) with confidence intervals, effect sizes, degrees of freedom and *P* value noted<br>*Give P values as exact values whenever suitable.*
☒	☐	For Bayesian analysis, information on the choice of priors and Markov chain Monte Carlo settings
☒	☐	For hierarchical and complex designs, identification of the appropriate level for tests and full reporting of outcomes
☐	☒	Estimates of effect sizes (e.g. Cohen's *d*, Pearson's *r*), indicating how they were calculated

Our web collection on statistics for biologists contains articles on many of the points above.

Software and code

Policy information about availability of computer code

Data collection	Leica Application Suite X (LAS X) was used to collect confocal images.
Data analysis	A description of all software used in data analysis is included in the Methods section. LAS X was used for HyVolution of confocal images and calculation of colocalization % and Pearson's R. FIJI's SpotCounter plugin (found on GitHub) was used to count spots of Crb, Uif, and Serp. R was used to generate boxplots.

For manuscripts utilizing custom algorithms or software that are central to the research but not yet described in published literature, software must be made available to editors/reviewers. We strongly encourage code deposition in a community repository (e.g. GitHub). See the Nature Research guidelines for submitting code & software for further information.

Data

Policy information about availability of data

All manuscripts must include a data availability statement. This statement should provide the following information, where applicable:

- Accession codes, unique identifiers, or web links for publicly available datasets
- A list of figures that have associated raw data
- A description of any restrictions on data availability

All data generated or analyzed during this study are included in this published article and its supplementary information files.

Field-specific reporting

Please select the one below that is the best fit for your research. If you are not sure, read the appropriate sections before making your selection.

☒ Life sciences ☐ Behavioural & social sciences ☐ Ecological, evolutionary & environmental sciences

For a reference copy of the document with all sections, see nature.com/documents/nr-reporting-summary-flat.pdf

Life sciences study design

All studies must disclose on these points even when the disclosure is negative.

Sample size	Sample-size was determined by the yield of available embryos, to a minimum of 3 for genotypes of which mutations of interest caused decreased embryonic viability. These sample sizes should be sufficient because all flies and embryos are raised in the same environment and have the same genetic background aside from the mutation of interest, which minimizes non-specific effects. Sample size was not pre-determined.
Data exclusions	No data were excluded from the analyses.
Replication	Experiments in this study were replicated through repeated testing on individual embryos (between 3-10 per genotype, depending on the assay). The results presented in the manuscript are all drawn from the analyses of multiple embryos. Specifics regarding the number of embryos assayed are presented clearly in the text and/ or in figure legends.
Randomization	Samples were allocated into experimental groups by genotype.
Blinding	Investigators were not blinded during data collection or analysis.

Reporting for specific materials, systems and methods

We require information from authors about some types of materials, experimental systems and methods used in many studies. Here, indicate whether each material, system or method listed is relevant to your study. If you are not sure if a list item applies to your research, read the appropriate section before selecting a response.

Materials & experimental systems

n/a	Involved in the study	
☐	☒	Antibodies
☒	☐	Eukaryotic cell lines
☒	☐	Palaeontology
☐	☒	Animals and other organisms
☒	☐	Human research participants
☒	☐	Clinical data

Methods

n/a	Involved in the study	
☒	☐	ChIP-seq
☒	☐	Flow cytometry
☒	☐	MRI-based neuroimaging

Antibodies

Antibodies used

Primary antibodies:
DrICECST9478: Rabbit anti-cleaved Drosophila ICE Asp230 (1:100) Cell Signaling Technology (CST) 9478S, lot #1
DrICECST13085S: Rabbit anti- Drosophila ICE (1:100) Cell Signaling Technology (CST) 13085S, lot #1
Mouse anti-Crumbs extracellular domain (1:25) Developmental Studies Hybridoma Bank (DSHB) at the University of Iowa
Mouse anti-Rab5 (1:100) BD Biosciences 610281, lot 6210957
Mouse anti-Rab7 (1:50) Developmental Studies Hybridoma Bank (DSHB) at the University of Iowa
Mouse anti-Rab11 (1:100) Fisher Scientific BDB610656, lot 6302832
Rabbit anti-Cleaved Caspase-3 (1:100) CST, Lot #42
Rabbit anti-Kune-Kune (Nelson et al., 2010)
Mouse anti-2A12 (1:1)
Guinea pig anti-Uninflatable (1:800) Generous gift of Rob Ward
Rabbit anti-Serp (1:400) Generous gift of Stefan Luschnig
Guinea-pig anti-DrICE (SK31) Generous gift of Pascal Meier
Rat anti-Clathrin (Chc) (1:40) Generous gift of Matthias Behr
Rabbit anti-Discs Large (Dlg) (1:500) Generous gift of Cho lab

Secondary antibodies
Goat anti-mouse IgG (H+L) highly cross-adsorbed secondary antibody Alexa Fluor 488 Plus Life Technologies A32723, lot SD250290
Goat anti-mouse IgM (H+L) highly cross-adsorbed secondary antibody Alexa Fluor 488 Life Technologies A10680, lot 1917945
Goat anti-rabbit IgG (H+L) highly cross-adsorbed secondary antibody Alexa Fluor Plus 647 Life Technologies A32733, lot RJ243415

Goat anti-guinea pig IgG (H+L) highly cross-adsorbed secondary antibody Alexa Fluor 568 Life Technologies A11075
IRDye 800CW Goat anti-Rabbit 0.5 mg Li-Cor 926-32211

Validation

DrICECST9478: Rabbit anti-cleaved Drosophila ICE Asp230 (1:100) Cell Signaling Technology (CST) 9478S, lot #1
- This antibody was validated for use in Drosophila by CST. Western blots with this antibody performed by CST have appropriate expected band sizes, etc. In this manuscript, we have validated this antibody for immunofluorescence through expected elimination of signal in a DrICE null mutant (Fig 2 h-h" and Fig S2 e, g).

DrICECST13085S: Rabbit anti- Drosophila ICE (1:100) Cell Signaling Technology (CST) 13085S, lot #1
- This antibody was validated for use in Drosophila by CST. Western blots with this antibody performed by CST have appropriate expected band sizes, etc.

Mouse anti-Crumbs extracellular domain (1:25) Developmental Studies Hybridoma Bank (DSHB) at the University of Iowa
- This antibody was generated and validated in the initial publication (Tepass and Knust, "Crumbs and Stardust act in a genetic pathway that controls the organization of epithelia in Drosophila melanogaster", Dev Biol 1993). Furthermore, in our experiments, we see signal in the expected pattern of Crumbs expression: all epithelia of ectodermal origin including the trachea, where we see the previously observed changes in Crumbs localization and abundance from stages 13-16 (Fig S1 b'-k')

Mouse anti-Rab5 (1:100) BD Biosciences 610281, lot 6210957
- This antibody was generated using purified human Rab5, which has a 75% amino acid identity to Drosophila Rab5. It has previously been used in immunofluorescence in Drosophila tissues (including, but not limited to: Fig. 8B: Woolworth et al, The Drosophila metastasis suppressor gene Nm23 homolog, awd, regulates epithelial integrity during oogenesis, Mol. Cell. Biol., 2009). In our manuscript, we observe the expected punctate and cytoplasmic pattern of Rab5 localization and expression.

Mouse anti-Rab7 (1:50) Developmental Studies Hybridoma Bank (DSHB) at the University of Iowa
- This antibody was validated for use in Drosophila immunofluorescence in the publication Riedel et al, An antibody toolkit for the study of membrane traffic in Drosophila melanogaster, Biology Open, 2016. In our manuscript, we observe the expected punctate and cytoplasmic pattern of Rab7 localization and expression.

Mouse anti-Rab11 (1:100) Fisher Scientific BDB610656, lot 6302832
- This antibody was generated using purified human Rab11, which has a 86% amino acid identity to Drosophila Rab11. In our manuscript, we observe the expected punctate and cytoplasmic pattern of Rab11 localization and expression.

Rabbit anti-Cleaved Caspase-3 (1:100) CST#9661, Lot #42
- This antibody was generated using purified human caspase-3, which has a 42% amino acid identity to Drosophila DrICE and 40% to Drosophila Dcp1. This antibody has been used many times in Drosophila tissues to detect apoptotic cells via mmunofluorescence (including, but not limited to, Fig. 4 E-F in Baer et al, "The role of apoptosis in shaping the tracheal system in the Drosophila embryo", Mech. Dev. 2010) In our manuscript, we observe the expected pattern of Caspase-3 homolog localization and expression, including high signal in apparently extruded cells in the DIAP1 null background, where derepressed caspase activity leads to increases in cell death (Fig. S2a, Fig 1m).

Rabbit anti-Kune-Kune (Nelson et al., 2010)
- This antibody was generated in our lab and validated for use in Drosophila immunofluorescence in Nelson et al., "The Drosophila claudin Kune-kune is required for septate junction organization and tracheal tube size control" Genetics, 2010. This tube of antibody is the same one used for the experiments in that paper. In our manuscript, we observed the expected pattern of Kune-kune localization at epithelial septate junctions.

Mouse anti-2A12 (1:1)
- This antibody was developed and validated as a marker for tracheal lumen in Beitel & Krasnow, Genetic control of epithelial tube size in the drosophila tracheal system, Development, 2000. The tube of antibody used in the present study was generated by Greg Beitel upon the initial publication and has been stored in aliquots at -80C. In our manuscript, we observed the expected pattern of 2A12 localization within the lumen of the trachea (Fig 1a, Fig 4j).

Guinea pig anti-Uninflatable (1:800) Generous gift of Rob Ward
- This antibody was generated and validated in Zhang and Ward, Uninflatable encodes a novel ectodermal apical surface protein required for tracheal inflation in Drosophila, Dev. Biol. 2009. In our manuscript, we observed the expected pattern of Uninflatable localization at the apical surface of epithelia (Fig. 2b').

Rabbit anti-Serp (1:400) Generous gift of Stefan Luschnig
- This antibody was generated and validated in Luschnig et al, serpentine and vermiform encode matrix proteins with chitin binding and deacetylation domains that limit tracheal tube length in Drosophila, Current Biology 2006. In our manuscript, we observed the expected pattern of Serp localization in the lumen of the trachea (Fig. 4j).

Guinea-pig anti-DrICE (SK31) Generous gift of Pascal Meier
- This antibody was generated and validated in Ditzel et al, Inactivation of effector caspases through nondegradative polyubiquitylation, Mol Cell, 2009. In our manuscript, we observed the expected diffuse cytopasmic pattern of SK31 in the embryo, per Ditzel et al, Fig 5C.

Rat anti-Clathrin (Chc) (1:40) Generous gift of Matthias Behr
-- This antibody was generated and validated in Wingen et al, Expression and localization of clathrin heavy chain in drosophila melanogaster, Gene Expr. Patterns, 2009. In our manuscript, we observed the expected pattern of Chc localization in trachea and elsewhere (Fig. 4j).

Rabbit anti-Discs Large (Dlg) (1:500) Generous gift of Cho lab
- This antibody was generated and validated in Woods et al, Dlg protein is required for junction structure, cell polarity, and proliferation control in Drosophila epithelia, JCB, 1996. In our manuscript, we observed the expected pattern of Dlg localization at the basolaterally located septate junctions of all epithelia including the trachea (Fig 3g).

Animals and other organisms

Policy information about studies involving animals; ARRIVE guidelines recommended for reporting animal research

Laboratory animals	The following mutant alleles were used in this paper: thJ5C8 (Hay et al., 1995), yorkieB5 (Silva et al., 2006), DrICE17 (Xu et al., 2006), DrICE[delta1] (Muro et al., 2006), shrub4 (Sweeney et al., 2006), and yurt65A (Laprise et al., 2006). DrICE overexpression in the trachea was achieved using the UAS/Gal4 system(Brand and Perrimon, 1993). The btl>Gal4 driver was used to express Gal4 in all tracheal cells; btl>Gal4 flies were crossed to UAS-DrICE flies, which had a transgenic DrICE cDNA downstream of UAS (see Transgenic Constructs below). The double mutant yurt65A, DrICE17 was generated using standard recombination crosses. Each single male resulting from the recombination crosses was screened for yurt65A via failure to a complement yurt65A single mutant and screened for the presence of the DrICE17 mutation by sequencing to identify the point mutation N116Y (Xu et al., 2006).
Wild animals	Study did not involve wild animals.
Field-collected samples	Study did not involve field-collected animals.
Ethics oversight	No ethical oversight was required as no vertebrate animals were involved in the research, and no ethical oversight of the experiments was required by our institution.

Note that full information on the approval of the study protocol must also be provided in the manuscript.
